# Emerging Trends in Pain Modulation by Metabotropic Glutamate Receptors

**DOI:** 10.3389/fnmol.2018.00464

**Published:** 2019-01-04

**Authors:** Vanessa Pereira, Cyril Goudet

**Affiliations:** IGF, CNRS, INSERM, Univ. de Montpellier, Montpellier, France

**Keywords:** pain, GPCR (G-protein-coupled receptors), receptor, glutamate (Glu), neurotransmitter, chronic pain, pharmacology, neuromodulation

## Abstract

Pain is an essential protective mechanism meant to prevent tissue damages in organisms. On the other hand, chronic or persistent pain caused, for example, by inflammation or nerve injury is long lasting and responsible for long-term disability in patients. Therefore, chronic pain and its management represents a major public health problem. Hence, it is critical to better understand chronic pain molecular mechanisms to develop innovative and efficient drugs. Over the past decades, accumulating evidence has demonstrated a pivotal role of glutamate in pain sensation and transmission, supporting glutamate receptors as promising potential targets for pain relieving drug development. Glutamate is the most abundant excitatory neurotransmitter in the brain. Once released into the synapse, glutamate acts through ionotropic glutamate receptors (iGluRs), which are ligand-gated ion channels triggering fast excitatory neurotransmission, and metabotropic glutamate receptors (mGluRs), which are G protein-coupled receptors modulating synaptic transmission. Eight mGluRs subtypes have been identified and are divided into three classes based on their sequence similarities and their pharmacological and biochemical properties. Of note, all mGluR subtypes (except mGlu6 receptor) are expressed within the nociceptive pathways where they modulate pain transmission. This review will address the role of mGluRs in acute and persistent pain processing and emerging pharmacotherapies for pain management.

## Introduction

Acute pain is an important protective function, detecting harmful stimuli and preventing body damage. However, chronic pain persists for a long time after the initial affliction, losing its role as a warning signal and must be considered as a disease *per se*. Patients suffering from chronic pain not only experience exacerbated responses to both painful (hyperalgesia) and non-painful stimuli (allodynia) (Sandkühler, 2009) but also frequently express emotional and cognitive impairments often resulting in anxiety and depression (McWilliams et al., 2003; Moriarty et al., 2011; Bushnell et al., 2013).

**Figure 1:**
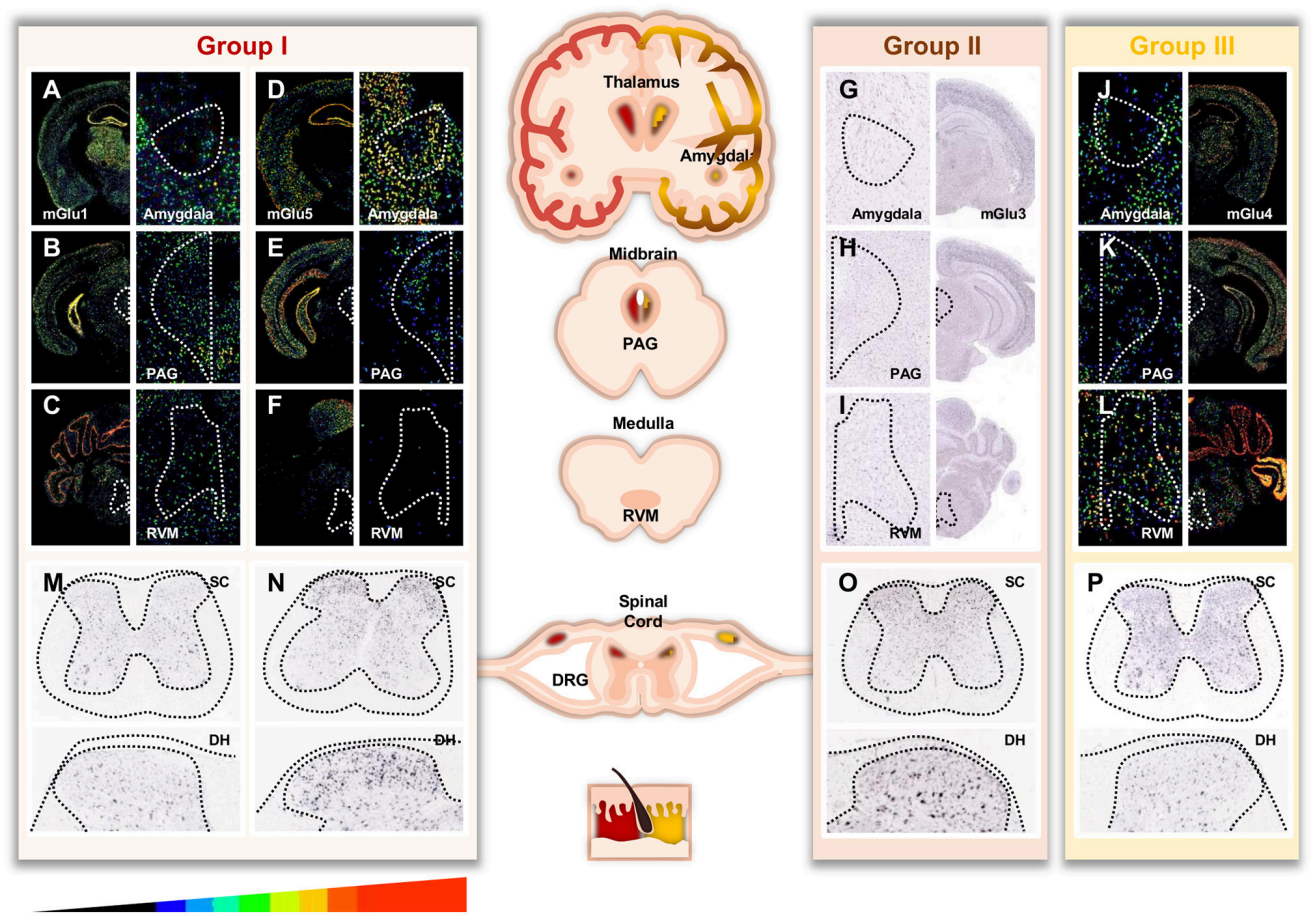
Distribution of mGluRs throughout important areas involved in pain. For **(A-F, J-L)** pictures, masks with pseudo colors were used to color scale the relative expression level of mGluR transcripts across sections (scale displayed at the bottom of the figure). For **(G-I, M-P)**, no expression filter was applied to recolour the ISH pictures. Image credit: Allen Institute. Masked ISH images of mGlu1 **(A)** and mGlu5 **(B)** transcripts in mice coronal section, notably in Thalamus and Amygdala. CeA (central nucleus of the amygdala) is magnified in the right panels (white dotted line, drawn according to the Allen Brain Atlas). Distribution of mGlu1 **(B,C)** and mGlu5 **(E,F)** mRNA in mice midbrain and medulla sections involved in descending modulation of pain. Magnification of the periaqueductal gray (PAG) and rostro ventral medulla (RVM) areas are shown in the right panels (white dotted line, drawn according to the Allen Brain Atlas). ISH images of mGlu3 **(G)** transcript in mice coronal section, notably in Thalamus and Amygdala. CeA is magnified in the left panel (white dotted line). Distribution of mGlu3 **(H,I)** mRNA in mice midbrain and medulla. Magnification of the PAG and RVM nucleus are shown in the left panels (white dotted line). Masked ISH images of mGlu4 **(J)** transcript in mice coronal section, notably in Thalamus and Amygdala. CeA is magnified in the left panel (white dotted line). Distribution of mGlu4 **(K,L)** mRNA in mice midbrain and medulla. Magnification of the PAG and RVM nucleus are shown in the left panels (white dotted line). Images are available for mGlu1 receptor (GMR1 gene) at http://mouse.brain-map.org/experiment/show/79591723, for mGlu5 receptor (GRM5 gene) at http://mouse.brain-map.org/experiment/show/73512423, for mGlu3 receptor (GMR3 gene) at http://mouse.brain-map.org/experiment/show/539, and for mGlu4 receptor (GRM4 gene) at http://mouse.brain-map.org/experiment/show/71247631. Distribution of mGlu1 **(M)**, mGlu5 **(N)**, mGlu3 **(O)**, mGlu4 **(P)** transcripts in mice spinal cord. Bottom panels are magnification of the dorsal horn. Images are available for mGlu1 at http://mousespinal.brain-map.org/imageseries/show.html?id=100036413, for mGlu5 receptor at http://mousespinal.brain-map.org/imageseries/show.html?id=100033614, for mGlu3 receptor at http://mousespinal.brain-map.org/imageseries/show.html?id=100039062 and for mGlu4 receptor at http://mousespinal.brain-map.org/imageseries/show.html?id=100018200.

Glutamate is the main excitatory neurotransmitter in the nervous system of adult mammals. Among the neurotransmitters involved in pain transmission from the periphery to the brain, glutamate has a leading role. Glutamate is also involved in central sensitization, which is associated with chronic pain. Glutamate action is mediated through ionotropic and metabotropic receptors. Ionotropic glutamate receptors (iGluRs) are ligand-gated ion channels involved in the fast synaptic response to glutamate. Metabotropic glutamate receptors (mGluRs) are G protein-coupled receptors that are responsible for the slow neuromodulatory response to glutamate. Eight mGluRs have been identified so far. They are named mGlu1 to mGlu8 receptors by chronological order of discovery. Later, based on their sequence homology, signalization and pharmacology, they were subdivided in three groups. Group I mGluRs (mGlu1 and 5) are canonically coupled to Gαq/11 and lead to phospholipase C (PLC) activation that promotes neuronal excitability and are mostly expressed postsynaptically. In contrast, group II (mGlu2 and 3) and group III (mGlu4, 6, 7, and 8) mGluRs are predominantly coupled to Gαi/o triggering adenylate cyclase (AC) inhibition. Group II and III mGluRs also regulate neuronal excitability and synaptic transmission through Gβγ subunits, which notably inhibit voltage-sensitive calcium channels and activate potassium channels. Both group II and group III mGluRs are mainly localized on presynaptic terminals. Both iGluRs and mGluRs (except mGlu6 receptor) are expressed all along the pain neuraxis where they shape the transmission of pain information (Figure 1). They are also involved in the induction and the maintenance of central sensitization of the pain pathway (Latremoliere and Woolf, 2009). This phenomenon is associated with hyperexcitability of the glutamatergic system which leads to the development of the main sensory symptoms observed in persons suffering from chronic pain.

Acting on the molecular mechanisms of glutamatergic transmission may, therefore, be a way of developing future analgesics counteracting chronic pain. However, even if iGluR selective antagonists have proven efficacious in releasing several pain states, drastically inhibiting glutamatergic transmission via iGluR blocking inevitably induces numerous side effects, notably hallucinations, ataxia and sedation (Bleakman et al., 2006). Therefore, the strategy of pharmacological modulation of mGluRs for the treatment of pain has been favored and significant effort has been devoted to better understanding the expression, the function and the role of these receptors in pain processing. The present review will focus on the role of mGluRs in acute and chronic pain at different levels–from the periphery to higher brain center involved in the perception and modulation of pain–and report the recent advances in the pharmacological strategy used to achieve mGluRs modulation.

## Pharmacology of mGluRs

Both orthosteric and allosteric ligands are available for pharmacological manipulation of mGluRs. Given their different binding sites, orthosteric ligands and allosteric modulators have specific pharmacological properties.

Orthosteric ligands are binding in the same pocket than the natural ligand (the orthosteric pocket). They are also referred to as competitive ligands. In mGluRs, the glutamate-binding pocket is located in the extracellular domain of the receptor. Due to the high degree of conservation of the glutamate-binding pocket among the mGluRs, the identification of subtype selective ligands is highly challenging. Therefore, many orthosteric ligands are selective for a specific group but do not discriminate between receptors within the group. The typical specific group I, II or III mGluRs agonists are S-3, 5-DHPG, LY379268 and L-AP4, respectively, and have been used in many preclinical studies. Recently, selective orthosteric ligands have been generated, LY2794193 for mGlu3 receptor (Monn et al., 2015, 2018) and LSP4-2022 for mGlu4 receptor (Goudet et al., 2012). They bind to residues of the orthosteric site and to specific residues and pockets surrounding the glutamate-binding pocket. LSP4-2022 has notably been used in several pain studies.

Allosteric modulators regulate the activity of a receptor by binding at a site distinct from the orthosteric site of endogenous ligands. In mGluRs, the binding site for most synthetic allosteric modulators which has been identified so far is located in the seven transmembrane domain. Interestingly, this pocket is less well conserved between the different receptors of the family, allowing the discovery of subtype selective ligands. Allosteric modulators may inhibit (negatively modulate) or potentiate (positively modulate) the activity of a co-binding orthosteric ligand at a target receptor and so can act as negative or positive allosteric modulators, respectively. Moreover, neutral allosteric ligands capable of inhibiting the action of either positive or negative allosteric modulators but devoid of activity by themselves have also been described (also referred to as silent allosteric modulators, SAM). Negative allosteric modulators (NAM) act as non-competitive antagonists and can have inverse agonist properties, meaning that they can inhibit the constitutive activity of the receptor. Interestingly, due to their non-competitive mode of action, the action of NAMs is less dependent on the concentration of endogenous ligands. Positive allosteric modulators (PAM) can enhance either the potency or the efficacy, or both, of orthosteric agonists. Therefore, in contrast to agonists that maintain the receptor active, pure PAMs potentiate the cellular response resulting from the action of the endogenous ligand. Some PAMs can also directly activate the receptor, referred to as agoPAMs, although such activity is usually partial.

The first described allosteric modulators of mGluRs were CPCCOEt, BAY36-7620 and MPEP, which display inverse agonist activity on mGlu1 and mGlu5 receptors (Litschig et al., 1999; Pagano et al., 2000; Carroll et al., 2001). Shortly after, a series of PAMs of mGlu1 receptors were described (Knoflach et al., 2001). To date, PAMs and NAMs have been described for most mGluRs [see (Lindsley et al., 2016) for a review] and have proven to be useful in exploring the function of mGluRs in pain.

Photopharmacology is a recent advance in the field of mGluRs. It is based on freely diffusible, light-operated ligands to control the function of the ligand on its target by light. Contrary to optogenetics, neither genetic modification of the targeted receptor nor exogenous expression are required, enabling the photocontrol of endogenous receptors. Two types of drugs have been developed for photopharmacology: photoactivable and photoswitchable ligands (Goudet et al., 2018). It allows the pharmacological manipulation of mGluRs with high spatial and temporal precision and holds great promise for exploring their physiological and pathological functions, notably in pain (Font et al., 2017; Gómez-Santacana et al., 2017; Zussy et al., 2018).

## Pain Modulation Following Systemic Administration of mGluRs Ligands

Since mGluRs are extensively expressed along the pain neuraxis (Figure 1), several preclinical studies have been performed to evaluate the impact of mGluRs ligands on pain following systemic administration (Tables 1–3). These preclinical studies outline the role of these different receptors on the regulation of pain. Additional studies have been performed to explore the role of these receptors at precise locations of the pain pathways and will be described in the following paragraphs.

**Table 1:** Pain modulation following systemic administration of group I mGluRs ligands.

**Receptor subtype**	**Drugs type**	**Name**		**ModelsSpecies**		**Effects Tests**	**References**
**Group I**							
**∙ mGlu1**	**NAM**	**FTIDC**		NaïveMice	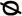	- No effect in thermal threshold - Tail immersion test	Satow et al., 2008
		**EMQMCM**		NaïveRats	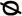	- No effect in thermal threshold - Radiant heat source	Sevostianova and Danysz, 2006
		**A-841720**	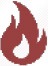	CFARats	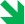	- Dose dependent increase of withdrawal latencies - Radiant heat source	El-Kouhen et al., 2006
		**LY456236**	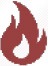	FormalinMice	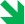	- Dose dependent decrease of pain-related behavior - Licking and flinching	Varty et al., 2005
		**EMQMCM**	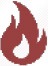	FormalinRats	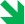	- Reduced manifestation of both phases - No development of tolerance - Licking behavior	Sevostianova and Danysz, 2006
		**FTIDC**	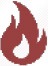	FormalinMice	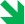	- Inhibit formalin-induced nociceptive behavior - Licking behavior	Satow et al., 2008
		**A-841720**	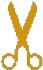	Skin incisionRats	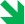	- Attenuation of spontaneous post-operative pain behavior - Significant motor side effects - Weight-bearing/Open field/Rotarod	Zhu et al., 2008
		**A-794282**	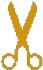	Skin incisionRats	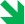	- Attenuation of spontaneous post-operative pain behavior - Significant motor side effects - Weight-bearing/Open field/Rotarod	Zhu et al., 2008
		**A-841720**	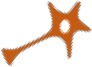	CCIRats	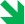	- Decrease mechanical allodynia - Motor and cognitive side effects at analgesic doses - Von frey	El-Kouhen et al., 2006
		**LY456236**	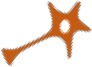	SNLRats	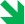	- Dose dependent increase of withdrawal threshold - Von Frey	Varty et al., 2005
		**A-841720**	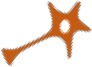	SNLRats	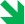	- Decrease mechanical allodynia - Motor and cognitive side effects at analgesic doses - Von frey	El-Kouhen et al., 2006
**∙ mGlu5**	**NAM**	**MPEP**		NaïveRats	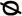	- No effect in thermal threshold - Radiant heat source	Sevostianova and Danysz, 2006
		**MTEP**		NaïveRats	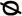	- No effect in thermal threshold - Radiant heat source	Sevostianova and Danysz, 2006
		**MPEP**	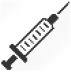	AIWMice	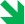	- Dose-dependent reduction of writhing activity - Number of cramps	Zhu et al., 2004
		**MPEP**	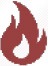	CarrageenanRats	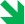	- Reversal of inflammatory hyperalgesia - Absence of locomotor side effects - Paw pressure/Rotarod assay	Walker et al., 2001a,b
		**MPEP**	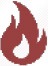	CarrageenanRats	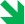	- Decrease thermal hyperalgesia without affecting paw oedema - Radiant heat source	Zhu et al., 2004
		**MPEP**	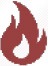	CFARats	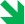	- Reversal of mechanical hyperalgesia - Paw pressure	Walker et al., 2001a,b
		**MPEP**	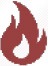	CFARats	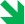	- Dose-dependent reversal of thermal and mechanical hyperalgesia - Paw pressure test/Radiant heat source	Zhu et al., 2004
		**Fenobam**	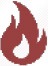	CFAMice	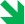	- Reduce thermal hypersensitivity - Increase in spontaneous locomotor activity, no effect in motor coordination - Radiant heat source/Open field/Rotarod	Montana et al., 2009
		**MPEP**	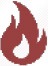	FormalinRats	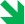	- Reduce phase I and II - Paw flinches	Zhu et al., 2004
		**MPEP**	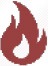	FormalinMice	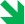	- Dose dependent decrease of pain-related behavior - Licking and flinching	Varty et al., 2005
		**MTEP**	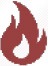	FormalinMice	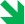	- Dose dependent decrease of pain-related behavior - Licking and flinching	Varty et al., 2005
		**MPEP**	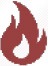	FormalinRats	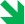	- Reduce the manifestation of both phases - Licking behavior	Sevostianova and Danysz, 2006
		**MPEP**	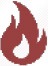	FormalinRats	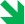	- Reduce the manifestation of both phases - Development of tolerance - Licking behavior	Sevostianova and Danysz, 2006
		**MPEP**	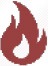	FormalinMice	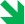	- Inhibit formalin-induced nociceptive behavior - Licking behavior	Satow et al., 2008
		**Fenobam**	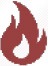	FormalinRats	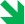	- Prevent formalin-induced spontaneous pain-related behavior - Licking, lifting, or flicking	Jacob et al., 2009
		**Fenobam**	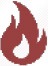	FormalinMice	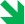	- Prevent formalin-induced spontaneous pain-related behavior - Licking, lifting, or flicking	Montana et al., 2009
		**Fenobam**	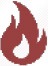	FormalinMice	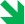	- Both acute and chronic treatment reduce phase I and II - No tolerance, increase in exploratory behavior, no impact in motor coordination - Licking behavior, Open field, Elevated O maze	Montana et al., 2011
		**MPEP**	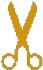	Skin incisionRats	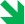	- Reduce post-operative pain - Von Frey/Radiant heat source	Zhu et al., 2004
		**MPEP**	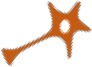	CCIRats	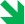	- Dose-dependent reversal of mechanical allodynia - Von Frey	Zhu et al., 2004
		**Fenobam**	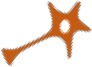	CCIRats	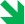	- No effect in mechanical allodynia - Electronic von Frey	Jacob et al., 2009
		**MPEP**	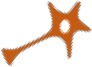	PSNSRats	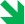	- No effect - Von Frey/Paw pressure test/Radiant heat source	Hudson et al., 2002
		**MPEP**	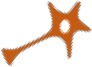	SNLRats	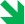	- No effect - Paw pressure	Walker et al., 2001a,b
		**MPEP**	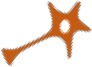	SNLRats	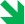	- Reverse thermal hyperalgesia - Fail to alter tactile allodynia or mechanical hyperalgesia - Von Frey/Paw pressure test/Radiant heat source	Hudson et al., 2002
		**MPEP**	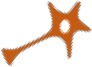	SNLRats	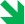	- Dose-dependent reversal of mechanical allodynia - Von Frey	Zhu et al., 2004
		**MPEP**	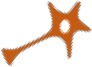	SNLRats	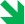	- Anxiolytic effect in naïve animals, reduce locomotor activity and coordination - Vogel conflict test	Varty et al., 2005
		**MTEP**	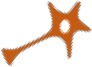	SNLRats	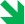	- Anxiolytic effect in naïve animals, reduce locomotor activity and coordination - Vogel conflict test	Varty et al., 2005
		**MPEP**	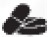	CIPNRats	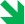	- Dose-dependent reversal of mechanical allodynia - Von Frey	Zhu et al., 2004

*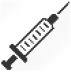 Symbols are used for model of pain induced by local injection, 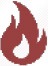 for inflammatory pain, 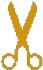 for post-operative pain, 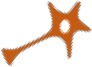 for neuropathic pain and for 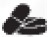 chemotherapy-induced neuropathic pain models. 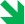, Decrease pain; 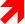, Increase pain; AIW, Acid-induced writhing; CCI, Chronic constriction injury; CFA, Complete Freund's Adjuvant; CIPN, Chemotherapy-induced peripheral neuropathy; PSNS, Partial sciatic nerve section; SNL, Spinal nerve ligation*.

### Group I mGluRs

Systemic administration of mGlu1 receptor antagonists are inefficient at altering normal pain threshold in naive animals (Maione et al., 1998; Sevostianova and Danysz, 2006). However, mGlu1 receptor inhibition relieves both mechanical and thermal hypersensitivity in various models of both inflammatory and neuropathic pain (Table 1) (Varty et al., 2005; El-Kouhen et al., 2006; Sevostianova and Danysz, 2006; Satow et al., 2008; Zhu et al., 2008). Similarly, systemic administration of mGlu5 receptor antagonists fails to modify basal thermal threshold (Sevostianova and Danysz, 2006), whereas it prevents mechanical and thermal hyperalgesia in a broad range of pain conditions from sub-chronic inflammatory pain to long lasting neuropathic pain (Table 1) (Walker et al., 2001a,b; Hudson et al., 2002; Zhu et al., 2004; Varty et al., 2005; Sevostianova and Danysz, 2006; Satow et al., 2008; Jacob et al., 2009; Montana et al., 2009; Zammataro et al., 2011). Of note, mGlu1 receptor inhibition induces motor and cognitive side effects at analgesic doses that could limit its use in clinical trials (El-Kouhen et al., 2006; Zhu et al., 2008). Consequently, mGlu5 receptor seems to be a better target to develop analgesic drugs. Although mGlu5 antagonists have been reported to induce tolerance and some locomotor deficits (Varty et al., 2005; Sevostianova and Danysz, 2006), it is interesting to point out that mGlu5 receptor antagonists reduce anxiety in naïve animals, a comorbidity often associated with chronic pain states (Varty et al., 2005).

### Group II mGluRs

Systematically administrated group II selective agonists have proven anti-hyperalgesic effects in both inflammatory and neuropathic pain without altering basal pain thresholds in healthy animals (Table 2) (Sharpe et al., 2002; Simmons et al., 2002; Satow et al., 2008; Johnson et al., 2017). Interestingly, selective group II mGluRs agonists have entered into clinical trials for the treatment of schizophrenia suggesting a safe profile of the drug in humans (Li et al., 2015; Muguruza et al., 2016).

**Table 2:** Pain modulation following systemic administration of group II mGluRs ligands.

**Receptor subtype**	**Drugs type**		**Name**	**ModelsSpecies**	**Effects Tests**		**References**
**Group II**							
**∙ mGlu2/3-selective**	**Agonist**	**LY379268**		NaïveRats	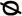	- No effects acute thermal nociceptive function - Tail flick test on Radiant heat source	Simmons et al., 2002
		**LY379268**		NaïveRats	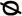	- No effects on withdrawal latencies to either mechanical or thermal stimulation - Paw pressure/Radiant heat source	Sharpe et al., 2002
		**LY2969822**	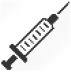	CAPRats	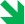	- Prevent tactile hypersensitivity - Oral prodrug of LY2934747 - Von Frey	Johnson et al., 2017
		**LY379268**	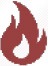	CarrageenanRats	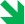	- Reduce inflammation induced hyperalgesia - Paw pressure/Radiant heat source	Sharpe et al., 2002
		**LY2969822**	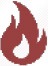	CFARats	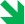	- Reduce pain related behavior - Oral prodrug of LY2934747 - Paw pressure	Johnson et al., 2017
		**LY354740**	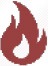	FormalinRats	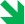	- Reduce pain related behavior - Licking behavior	Simmons et al., 2002
		**LY379268**	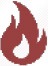	FormalinRats	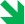	- Reduce pain related behavior - Reverse mGlu2/3 antagonist LY341495 - Licking behavior	Simmons et al., 2002
		**LY389795**	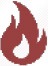	FormalinRats	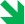	- Reduce pain related behavior - Licking behavior	Simmons et al., 2002
		**LY379268**	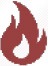	FormalinMice	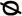	- No effect - Licking behavior	Satow et al., 2008
		**LY2934747**	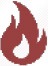	FormalinRats	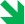	- Reduce pain related behavior - Blocked by LY341495 - Licking behavior	Johnson et al., 2017
		**LY379268**	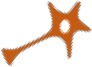	SNLRats	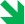	- Reverse mechanical allodynia - Von frey	Simmons et al., 2002
		**LY2934747**	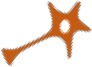	SNLRats	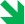	- Prevent tactile hypersensitivity - Von Frey	Johnson et al., 2017

*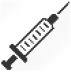 Symbols are used for model of pain induced by local injection, 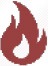 for inflammatory pain, 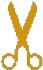 for post-operative pain, 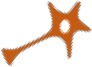 for neuropathic pain and 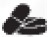 for chemotherapy-induced neuropathic pain models. 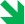, Decrease pain; 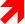, Increase pain; CAP, Capsaicin; CFA, Complete Freund's Adjuvant; SNL, Spinal nerve ligation*.

### Group III mGluRs

Only a few studies have investigated the effect of systemic administration of group III selective compounds in pain perception (Table 3). Systemic delivery of mGlu4 receptor agonist alleviates mechanical hypersensitivity provoked by carrageenan-induced inflammation (Vilar et al., 2013). AMN082, an mGlu7 receptor PAM prevents hyperalgesia in inflammatory models (Dolan et al., 2009). The same compound injected systematically reduces mechanical allodynia and thermal hyperalgesia induced by chronic constriction injury to the sciatic nerve and potentiates the effect of morphine (Osikowicz et al., 2008). This drug also exhibits antidepressant-like and anxiolytic-like effects (Bradley et al., 2012). In addition to the mGlu7 receptor, other mechanisms can contribute to these effects since the AMN082 compound is rapidly metabolized *in vivo* into a monoamine transporter inhibitor (Sukoff Rizzo et al., 2011). Surprisingly, systemically administrated mGlu7 receptor negative allosteric modulators (NAMs) also have anti-hyperalgesic effects in neuropathic pain models (Palazzo et al., 2015). As detailed further in this review, pharmacological activation of mGlu7 receptors can lead to opposite effects depending on the administration site. Neuropathic pain induces variation in mGlu7 receptor expression that could imbalance the pronociceptive and antinociceptive role of mGlu7 receptor (Osikowicz et al., 2009; Palazzo et al., 2013, 2015).

**Table 3:** Pain modulation following systemic administration of group III mGluRs ligands.

**Receptor subtype**	**Drugs type**	**Name**		**ModelsSpecies**	**Effects Tests**		**References**
**Group III**							
**mGlu4**	**Agonist**	**LSP4-2022**	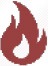	CarrageenanRats	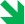	- Reduce mechanical hypersensitivity - Paw pressure	Vilar et al., 2013
**mGlu7**	**PAM**	AMN082^*^	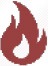	CarrageenanRats	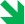	- Prevent thermal hyperalgesia (before carrageenan) and inhibit thermal hyperalgesia and mechanical allodynia - Radiant heat source/Dynamic plantar aesthesiometer	Dolan et al., 2009
		AMN082^*^	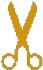	Skin incisionRats	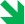	- Pre surgical and postsurgical administration inhibits thermal hyperalgesia, but not mechanical allodynia - Radiant heat source/Dynamic plantar aesthesiometer	Dolan et al., 2009
	**NAM**	**MMPIP**		SNIMice	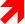	- Increase thermal and mechanical thresholds - Decrease anxiety-related behavior and improve cognitive performance - Radiant heat source/Dynamic plantar aesthesiometer/EPM/Tail suspension/Marble burying test.	Palazzo et al., 2015
		**XAP044**		SNIMice	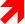	- Increase thermal and mechanical thresholds - Decrease anxiety-related behavior - Radiant heat source/Dynamic plantar aesthesiometer/EPM/Tail suspension/Marble burying test.	Palazzo et al., 2015
**mGlu8**	**Agonist**	**DCPG**	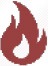	CarrageenanMice	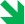	- Reduce carrageenan-induced thermal hyperalgesia and mechanical allodynia - Blocked by intra-PAG MSOP - Radiant heat source/Dynamic plantar aesthesiometer	Marabese et al., 2007
		**DCPG**	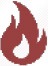	FormalinMice	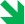	- Decrease both early and delayed nociceptive responses - Blocked by intra-PAG MSOP - Licking, lifting, or flicking	Marabese et al., 2007
		**DCPG**		CCIMice	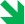	- Effective 3 days after surgery but ineffective in alleviating thermal hyperalgesia and mechanical allodynia 7 days after - Radiant heat source/Dynamic plantar aesthesiometer	Marabese et al., 2007

* *Of note, in vivo actions of AMN082 should be interpreted with caution because they may involve other mechanisms in addition to mGlu7. Indeed, an AMN082 metabolite can inhibit monoamine transporters Sukoff Rizzo et al., 2011*.

*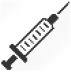 symbols are used for model of pain induced by local injection, 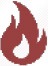 for inflammatory pain, 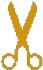 for post-operative pain, 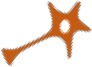 for neuropathic pain and 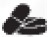 for chemotherapy-induced neuropathic pain models. 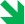, Decrease pain; 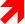, Increase pain; CCI, Chronic constriction injury; SNI, Spared nerve injury*.

Systemic delivery of a mGlu8 receptor agonist also decreases nociceptive responses in inflammatory and neuropathic models, which is inhibited by blocking group III mGluRs in the PAG (Marabese et al., 2007).

## Role of Metabotropic Glutamate Receptors in Peripheral Mechanisms of Sensory Transmission

Sensory transmission initiates with the detection by primary afferents in the periphery of a broad range of stimuli such as mechanical, thermal or chemical stimuli. Primary afferents are specialized neurons translating information detected at the periphery into electrical signals which are conveyed through their cell bodies located in the dorsal root ganglia (DRG) to their projections into the dorsal horn of the spinal cord. Spinal neurons then project to higher centers in the brain which process the sensory information. After nerve injury or inflammation, a number of dysregulations occur in sensory neurons affecting activity, properties or gene expression, driving an increased sensitivity to both non-noxious and noxious stimuli with or without ectopic activities. Because the primary afferents are the first relay of nociceptive transmission and can trigger the chronicization of pain, they represent an interesting target for the development of analgesic drugs.

Early evidence of a glutamate role in nociceptive transmission at the periphery derived from the observation of thermal and mechanical hypersensitivity following subcutaneous injection of glutamate into naive rat hind paw (Carlton et al., 1995; Jackson et al., 1995), first believed to be only triggered by iGluR activation (Zhou et al., 1996). Furthermore, in rodents, glutamate concentration rises in inflamed tissue (Omote et al., 1998) and after sciatic nerve stimulation (deGroot et al., 2000). Elevated levels of glutamate have also been measured in synovial fluid from knee joints of arthritis patients highlighting the clinical relevance of glutamate modulation as a peripheral mediator of pain perception (McNearney et al., 2000). Since then, an increasing number of studies have reported the involvement of mGluRs at the periphery.

Recently, a single-cell transcriptome analysis has reported the expression of mGluR transcripts in mice DRG. Among the most expressed are mGlu7, mGlu3, mGlu4, mGlu8, and mGlu5 receptors (Usoskin et al., 2015). Transcriptome analysis provides evidence for the expression of mGluRs in cell bodies but whether these receptors are expressed at the peripheral terminal, the spinal projection endings, or both, must be further investigated. mGluRs expression has also been reported in trigeminal ganglia, notably mGlu1, mGlu2/3, and mGlu8 receptors (Boye Larsen et al., 2014).

### Group I mGluRs

Group I mGlu1 and mGlu5 receptors are expressed in nociceptive afferents (Bhave et al., 2001; Walker et al., 2001a,b). Together with iGluR, group I mGluRs are involved in capsaicin induced glutamate release, a process that could contribute to nociceptive responses evoked by the TRPV1 agonist (Jin et al., 2009). Intraplantar injection of group I agonists in rodents enhances thermal sensitivity and reciprocally, peripherally applied group I antagonist reduced hyperalgesia in animal models of inflammatory or neuropathic pain (Table 4) (Dogrul et al., 2000; Bhave et al., 2001; Walker et al., 2001a,b). Application of mGlu5 receptor antagonist at peripheral afferent endings also reduces visceral nociception (Table 5) (Lindström et al., 2008). More recently, the analgesic potential of peripheral mGlu5 receptor blockade has been highlighted using an mGlu5 selective photoactivable NAM. Photoactivable ligands, also called caged-ligands, are constituted of a ligand linked to a photo-labile protecting group that will be removed following illumination, enabling the precise control of the onset of drug activity at a specific location (Goudet et al., 2018). Following systemic injection of the inactive caged-mGlu5 NAM, analgesia in both phases of the formalin test can be induced by local illumination in the paw (Table 5) (Font et al., 2017).

**Table 4:** Pain modulation following local administration of group I mGluRs ligands.

**Receptor subtype**	**Localization**	**Drugs type**	**Name**		**Models Species**	**EffectsTests**		**References**
**Group I**								
**∙ mGlu1/5 selective**	**Periphery**	**Agonist**	**RS-DHPG**		NaïveMice	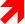	- Reduction of thermal withdrawal latency - Radiant heat source	Bhave et al., 2001
	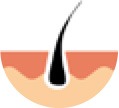		**RS-DHPG**		NaïveRats	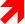	- Produce mechanical hyperalgesia - Paw pressure test	Walker et al., 2001a,b
			**RS-DHPG**		NaïveRats	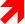	- Decrease the mechanical threshold to noxious stimulation of the masseter muscle - Prevented by MPEP but not CPCCOEt - Von Frey	Lee and Ro, 2007
			**S-DHPG**		NaïveRats	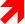	- Reduction of thermal withdrawal latency - Radiant heat source	Jin et al., 2009
			**RS-DHPG**		NaïveRats	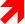	- Induce mechanical hyperalgesia in the masseter muscle - Attenuated by AMG9810, a specific TRPV1 antagonist - Von Frey	Chung et al., 2015
		**NAM**	**CPCCOEt**	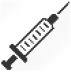	CAP injRats	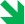	- Dose dependent increase of withdrawal latencies - Radiant heat source	Jin et al., 2009
	**Spinal cord**	**Agonist**	**RS-DHPG**		NaïveRats	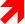	- Long lasting spontaneous nociceptive behaviors - Elevating, shaking, stamping of the hindpaw/elevating or whipping of the tail/liking or biting the tail	Fisher and Coderre, 1996
	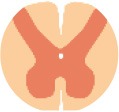		**RS-DHPG**		NaïveRats	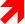	- Mechanical allodynia and hyperalgesia, thermal hyperalgesia - Von Frey/Tail clip/Hot plate	Fisher and Coderre, 1998
			**S-DHPG**		NaïveSheep	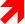	- Reduction of mechanical thresholds - Blocked by co-administration of the group I antagonist AIDA - Blunt pin	Dolan and Nolan, 2000
			**RS-DHPG**		NaïveMice	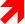	- Increase spontaneous nociceptive behavior - Licking of the flanks, tail, and hindpaws	Karim et al., 2001
			**RS-DHPG**		NaïveRats	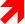	- Increase spontaneous nociceptive behavior - Blocked by MPEP - Licking of tail and hindpaws	Lorrain et al., 2002
			**RS-DHPG**		NaïveMice	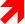	- Increase spontaneous nociceptive behavior - Blocked by MEK inhibitor U0126 - Licking, scratching and lifting behaviors	Adwanikar et al., 2004
			**RS-DHPG**		NaïveRats	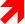	- Spontaneous nociceptive behaviors induction - Licking of the flanks, tail, and hindpaw	Hu et al., 2007
			**RS-DHPG**	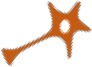	CCIRats	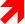	- Increase the hind paw frequency and duration of lifting - Blocked by MPEP - Cold plate	Hama, 2003
		**Antagonist**	**LY393053**	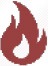	CFARats	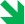	- Reduction of glutamate-induced spontaneous pain behaviors - Time spent licking	Vincent et al., 2017
			**S-4CPG**	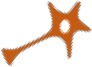	CCIRats	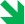	- Attenuation of mechanical allodynia and cold hyperalgesia - Von Frey/1 cm deep 1°C water bath	Fisher et al., 1998
			**LY393053**	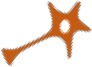	SNIRats	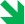	- Weak attenuation of glutamate-induced spontaneous pain behavior - Time spent licking the hind paws, lower legs or tail	Vincent et al., 2016
	**Amygdala**	**Agonist**	**DHPG**		NaiveMice	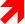	- Side dependent increase of mechanical hypersensitivity - Von Frey	Kolber et al., 2010
	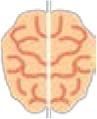		**DHPG**		NaïveRats	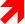	- Increased the duration of vocalizations - Decreased the hindlimb withdrawal threshold - Knee compression/colorectal distension	Li et al., 2011
	**PAG**	**Agonist**	**S-DHPG**		NaïveMice	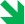	- Increase the latency of the nociceptive reaction - Hot plate	Maione et al., 1998
			**S-DHPG**	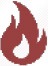	FormalinMice	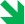	- Decrease phase II - Licking behavior	Maione et al., 2000
		**Antagonist**	**RS-AIDA**		NaïveMice	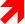	- Decrease the latency of the nociceptive reaction - Hot plate	Maione et al., 1998

*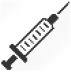 Symbols are used for model of pain induced by local injection, 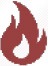 for inflammatory pain, 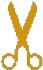 for post-operative pain, 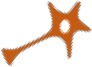 for neuropathic pain and 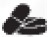 for chemotherapy-induced neuropathic pain models. 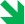, Decrease pain; 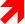, Increase pain; CAP, Capsaicin; CCI, Chronic constriction injury; CFA, Complete Freund's Adjuvant; SNI, Spared nerve injury*.

**Table 5:** Pain modulation following local administration of selective mGlu1 or mGlu5 ligands.

**Receptor subtype**	**Localization**	**Drugs type**	**Name**		**Models Species**	**EffectsTests**		**References**
**Group I**								
**∙ mGlu1**	**Periphery**	**Antagonist**	**LY367385**	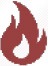	IL-1ß inj Rats	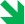	- Decrease IL-1b-induced mechanical allodynia in orofacial area - Air puff	Ahn et al., 2005
	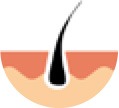	**NAM**	**CPCCOEt**	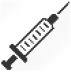	CAP inj Rats	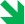	- Dose dependent increase of withdrawal latencies - Radiant heat source	Jin et al., 2009
			**CPCCOEt**	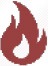	IL-1ß inj Rats	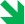	- Decrease IL-1b-induced mechanical allodynia in orofacial area - Air puff	Ahn et al., 2005
	**Spinal cord**	**Antagonist**	**RS-AIDA**	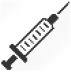	CAP inj Rats	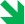	- Reduction of mechanical hypersensitivity, no effect in thermal hyperalgesia - Von Frey/Paw immersion	Soliman et al., 2005
	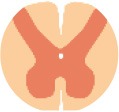		**RS-AIDA**	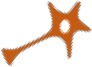	CCI Rats	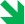	- Pretreatment produced reductions in the development of mechanical and cold hypersensitivity - Von Frey/1 cm deep 1°C water bath	Fisher et al., 2002
		**NAM**	**CPCCOEt**	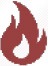	FormalinMice	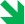	- Decrease phase II - Licking behavior	Karim et al., 2001
	**Amygdala**	**Antagonist**	**LY367385**		NaïveRats	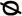	- No effect - Knee compression/colorectal distension	Li et al., 2011
		**NAM**	**CPCCOEt**	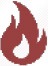	Carrageenan Rats	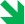	- Reduce mechanical hyperalgesia - Dynamic Plantar Aesthesiometer	Luongo et al., 2013
	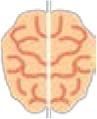		**CPCCOEt**	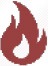	MA Rats	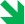	- Reduction of vocalizations induced by mechanical stimulation - Knee compression	Han and Neugebauer, 2005
	**Striatum**	**NAM**	**CPCCOEt**	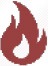	MA Rats	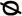	- No effect - Knee compression	Han and Neugebauer, 2005
**∙ mGlu5**	**Periphery**	**Agonist**	**CHPG**		NaïveRats	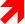	- Produced mechanical hyperalgesia - Paw pressure test	Walker et al., 2001a,b
	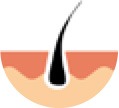	**NAM**	**MPEP**		NaïveRats	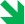	- Inhibit the visceromotor responses - Colorectal distension	Lindström et al., 2008
			**MPEP**	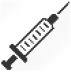	CAP inj Rats	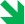	- Dose dependent increase of withdrawal latencies - Radiant heat source	Jin et al., 2009
			**MPEP**	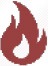	CFA Rats	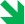	- Reduction of mechanical hyperalgesia - Paw pressure test	Walker et al., 2001a,b
			**MPEP**	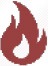	IL-1ß inj Rats	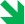	- Decrease IL-1b-induced mechanical allodynia in orofacial area - Air puff	Ahn et al., 2005
			**SIB1893**	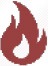	IL-1ß inj Rats	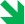	- Decrease IL-1b-induced mechanical allodynia in orofacial area - Air puff	Ahn et al., 2005
			**JF-NP-26 Photoactivable**	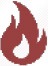	FormalinMice	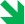	- Decrease both at phase I and phase II - Licking behavior	Font et al., 2017
			**MPEP**	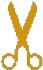	Skin incision Rats	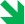	- Dose-dependent reduction of non-evoked pain - Weight-bearing	Zhu et al., 2005
			**SIB-1757**	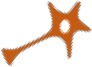	SNL Rats	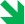	- No effect in acute pain - Reversal of thermal hyperalgesia - Von frey filaments/Radiant heat source	Dogrul et al., 2000
	**Spinal cord**	**Agonist**	**trans-ADA**		NaïveRats	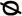	- No effect in spontaneous nociceptive behaviors - Elevating, shaking, stamping of the hindpaw/elevating or whipping of the tail/liking or biting the tail	Fisher and Coderre, 1996
	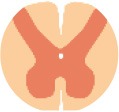	**NAM**	**MPEP**	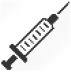	CAP inj Rats	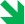	- Reduction of mechanical hypersensitivity, no effect in thermal hyperalgesia - Von Frey/Paw immersion	Soliman et al., 2005
			**Fenobam**	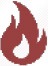	CFA Rats	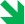	- Reduction of glutamate-induced spontaneous pain behaviors and rewarding under pain conditions - Time spent licking/CPP	Vincent et al., 2017
			**MPEP**	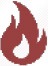	FormalinMice	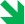	- Decrease phase II - Licking behavior	Karim et al., 2001
			**MPEP**	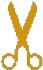	Skin incision Rats	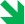	- Dose-dependent reduction of non-evoked pain - Weight-bearing	Zhu et al., 2005
			**MPEP**	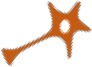	CCI Rats	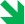	- Pretreatment produced reductions in the development of mechanical hypersensitivity (but not cold hypersensitivity) - Von Frey/1 cm deep 1°C water bath	Fisher et al., 2002
			**MPEP**	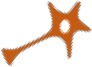	CCI Rats	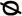	- No effect in cold threshold - Cold plate	Hama, 2003
			**Fenobam**	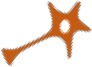	SNI Rats	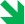	- Reduction of glutamate-induced spontaneous pain behaviors and mechanical allodynia - Time spent licking the hind paws, lower legs or tail/Von frey	Vincent et al., 2016
			**SIB-1757**	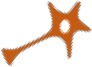	SNL Rats	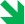	- No effect in acute pain - Reversal of thermal hyperalgesia and partial reversal of tactile allodynia - Frey filaments/Radiant heat source	Dogrul et al., 2000
			**SIB-1757**	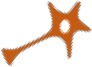	SNL Rats	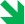	- No effect in acute pain - Reversal of thermal hyperalgesia and partial reversal of tactile allodynia - Frey filaments/Radiant heat source	Dogrul et al., 2000
			**MPEP**	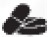	CIPN Rats	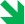	- Reversed pain hypersensitivity - Von Frey/Paw pressure test	Xie et al., 2017
	**Amygdala**	**NAM**	**MPEP**		NaïveRats	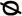	- No effect - Knee compression/colorectal distension	Li et al., 2011
	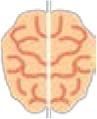		**MPEP**	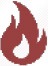	Carrageenan Rats	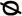	- No effect on mechanical hyperalgesia - Dynamic Plantar Aesthesiometer	Luongo et al., 2013
			**MPEP**	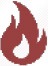	FormalinMice	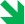	- Side dependent mechanical hypersensitivity reduction - Von Frey	Kolber et al., 2010
			**MPEP**	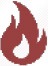	MA Rats	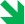	- Reduction of vocalizations induced by mechanical stimulation - Knee compression	Han and Neugebauer, 2005
			**Alloswitch-1** Photoswitchable	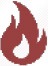	CFA Mice	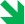	- Restore mechanical sensitivity - Von Frey	Gómez-Santacana et al., 2017
	**Thalamus**	**NAM**	**JF-NP-26** Photoactivable	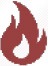	FormalinMice	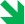	- Decrease both at phase I and phase II - Licking behavior	Font et al., 2017
			**JF-NP-26** Photoactivable	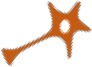	CCI Mice	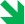	- Significantly increased pain thresholds - Von frey filaments	Font et al., 2017
	**Striatum**	**NAM**	**MPEP**	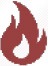	MA Rats	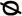	- No effect - Knee compression	Han and Neugebauer, 2005
	**Prefrontal cortex**	**NAM**	**MPEP**	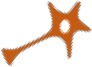	SNL Rats	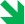	- Decrease tactile hypersensitivity and depressive-like behavior - Von Frey/Forced swimming test/Open field/Conditioned place preference	Chung et al., 2017

*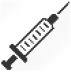 Symbols are used for model of pain induced by local injection, 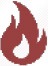 for inflammatory pain, 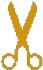 for post-operative pain, 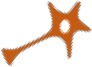 for neuropathic pain and 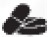 for chemotherapy-induced neuropathic pain models. 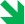, Decrease pain; 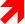, Increase pain; CAP, Capsaicin; CCI, Chronic constriction injury; CFA, Complete Freund's Adjuvant; CIPN, Chemotherapy-induced peripheral neuropathy; MA, Mono arthritis; SNL, Spinal nerve ligation; SNI, Spared nerve injury*.

### Group II mGluRs

Primary sensory neurons express mGlu2 and mGlu3 receptors in both peripheral terminals and dorsal horn projection (Carlton et al., 2001; Carlton and Hargett, 2007). In DRG, mGlu2/3 receptors are largely co-localized with TRPV1 channel (Carlton et al., 2009). Consistent with this co-expression, group II mGluR antagonists increase hyperalgesia evoked by capsaicin, a TRPV1 agonist, and this effect is blocked by group II mGluR agonists (Table 6) (Carlton et al., 2011). However, a recent report has demonstrated that mGlu2/3 receptors activation abolishes TRPV1 sensitization in mouse sensory neurons, but not in humans (Sheahan et al., 2018).

**Table 6:** Pain modulation following local administration of group II mGluRs ligands.

**Receptor subtype**	**Localization**	**Drugs type**	**Name**		**Models Species**	**Effects Tests**		**References**
**Group II**								
**∙mGlu2/3-selective**	**Periphery**	**Agonist**	**LY314582**		NaïveRats	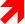	- Slight decrease of mechanical threshold - Paw pressure test	Walker et al., 2001a,b
	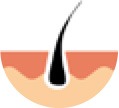		**APDC**		NaïveRats	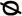	- No effect thermal withdrawal latency - Paw pressure test/Radiant heat source	Du et al., 2008
			**L-CCG-1**		NaïveRats	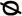	- No effect thermal withdrawal latency - Radiant heat source	Jin et al., 2009
			**APDC**	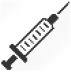	CAP injRats	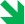	- Attenuate capsaicin-induced nociceptive behaviors - Flinching and lifting/licking	Carlton et al., 2009
			**APDC**	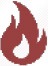	CarrageenanMice	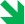	- Restore mechanical thresholds - Von Frey	Yang and Gereau, 2003
			**APDC**	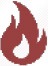	CarrageenanRats	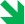	- Recovery of reduced weight load - Reduction of mechanical hyperalgesia - Von frey filaments/weight-bearing	Lee et al., 2013
			**APDC**	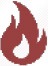	FormalinRats	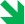	- Reduce flinching and L/L - Blocked by LY341495 - Flinching and lifting/licking	Du et al., 2008
			**APDC**	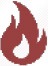	IL-1ß injRats	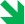	- Reduce IL-1b-induced mechanical allodynia - Inhibited by pretreatment with LY341495 - Air puff	Ahn et al., 2005
			**DCG-IV**	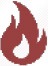	IL-1ß injRats	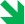	- Reduce IL-1b-induced mechanical allodynia - Inhibited by pretreatment with LY341495 - Air puff	Ahn et al., 2005
			**APDC**	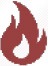	Inf soupRats	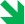	- Reduce heat and mechanical hyperalgesia - Radiant heat source/Von frey	Du et al., 2008
			**APDC**	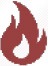	PGE2 injMice	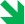	- Restore mechanical thresholds - Blocked by LY341495 - Von Frey	Yang and Gereau, 2003
		**Antagonist**	**MCCG**	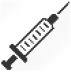	CAP injRats	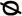	- No significant changes in withdrawal latencies - Radiant heat source	Jin et al., 2009
			**LY341495**	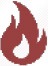	CarrageenanMice	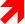	- Prolong mechanical allodynia - Von Frey	Yang and Gereau, 2003
			**LY341495**	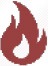	PGE2 injMice	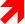	- Prolong PGE2-induced mechanical allodynia - Von Frey	Yang and Gereau, 2003
	**Spinal cord**	**Agonist**	**APDC**		NaïveRats	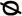	- No effect in spontaneous nociceptive behaviors - Elevating, shaking, stamping of the hindpaw/elevating or whipping of the tail/liking or biting the tail	Fisher and Coderre, 1996
	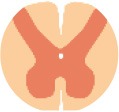		**APDC**		NaïveRats	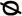	- No effect in spontaneous nociceptive behaviors - Elevating, shaking, stamping of the hindpaw/elevating or whipping of the tail/liking or biting the tail	Fisher and Coderre, 1996
			**L-CCG-I**		NaïveSheep	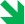	- Increase mechanical withdrawal thresholds - Blocked by EGLU - Blunt pin	Dolan and Nolan, 2000
			**DCG-IV**		NaïveRats	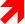	- Induce hyperalgesia - Paw pressure	Zhou et al., 2011
			**APDC**	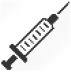	CAP injRats	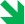	- Reduction of mechanical hypersensitivity, no effect in thermal hyperalgesia - Von Frey/Paw immersion	Soliman et al., 2005
			**APDC**	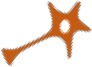	CCIRats	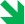	- Pretreatment produced reductions in the development of mechanical and cold hypersensitivity - Von Frey/1 cm deep 1°C water bath	Fisher et al., 2002
			**DCG-IV**	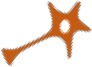	SNLRats	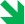	- Dose-dependent attenuation of allodynia and hyperalgesia - Von Frey/Paw pressure	Zhou et al., 2011
	**Thalamus**	**Antagonist**	**EGLU**	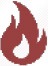	CFARats	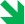	- Decrease pain behavior - Ankle-bend test	Neto and Castro-Lopes, 2000
	**PAG**	**Agonist**	**L-CCG-1**		NaïveMice	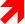	- Decrease the latency of the nociceptive reaction - Hot plate	Maione et al., 1998
	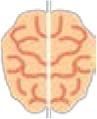		**L-CCG-1**	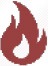	FormalinMice	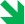	- Decrease phase II - Licking behavior	Maione et al., 2000
		**Antagonist**	**EGLU**		NaïveMice	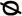	- No effect on nociceptive reaction - Hot plate	Maione et al., 1998

*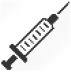 Symbols are used for model of pain induced by local injection, 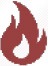 for inflammatory pain, 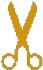 for post-operative pain, 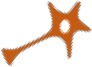 for neuropathic pain and 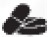 for chemotherapy-induced neuropathic pain models. 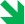, Decrease pain; 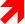, Increase pain; CAP, Capsaicin; CCI, Chronic constriction injury; CFA, Complete Freund's Adjuvant; Inf soup, Inflammatory soup; SNL, Spinal nerve ligation*.

In cultured DRG neurons, group II mGluRs also negatively regulate TTX resistant sodium channels (Yang and Gereau, 2004). Local administration of group II agonist in the knee joint both prevents and reduces carrageenan-induced arthritis (Lee et al., 2013). Due to the lack of selective compounds that can discriminate between mGlu2 and mGlu3 receptors, the individual contribution of those two receptors to pain modulation has remained unclear for a long time. However, the generation of mGlu2 and mGlu3 receptor knockout mice allowed the precise investigation of the role of each subtype in nociception and revealed a predominant role of the mGlu2 over mGlu3 receptor (Zammataro et al., 2011).

In line with the pharmacological evidence, mGlu2 receptor overexpression in DRG induces analgesia in models of inflammatory and neuropathic pain (Chiechio et al., 2002, 2009). L-acetylcarnitine, a drug known to enhance mGlu2 receptor expression in DRG through epigenetic mechanisms induces a long-lasting analgesia in both inflammatory and neuropathic pain models (Notartomaso et al., 2017). Strikingly, N-acetyl-cysteine, a drug enhancing mGlu2 receptor expression in rodents, reduces nociceptive transmission in humans (Truini et al., 2015). Moreover, in a recent report using cultured DRG neurons from both mice and humans, PGE2 evoked neuron hyperexcitability was blocked by group II mGluR activation (Davidson et al., 2016). This data suggests that activation of group II mGluRs leads to an analgesic effect in rodents and humans, making group II mGluRs an interesting target for development of peripherally active drugs for the treatment of chronic pain.

### Group III mGluRs

Most group III mGluRs are expressed in the pain pathway, except the mGlu6 receptor which is expressed mainly in the retina (Vardi et al., 2000). The presence of mGlu4, mGlu7, and mGlu8 receptors have been detected in DRG and trigeminal ganglia (Li et al., 1996; Azkue et al., 2001; Carlton and Hargett, 2007). The mGlu8 receptor is expressed in DRG and peripheral terminals where it is widely co-expressed with TRPV1. Intraplantar injection of group III agonists significantly reduced capsaicin evoked pain behavior (Table 7; Govea et al., 2012). Similar to group II agonists, local administration in the knee joint of group III mGluRs agonist provokes analgesia in carrageenan-induced arthritic pain model (Lee et al., 2013). Specific contribution of each subtype to the antinociceptive effect of broad range group III mGluRs need to be further investigated.

**Table 7:** Pain modulation following local administration of group III mGluRs ligands.

**Receptor subtype**	**Localization**	**Drugs type**	**Name**		**Models Species**	**Effects Tests**		**References**
**Group III**								
**∙pan-group III selective**	**Periphery**	**Agonist**	**L-AP4**		NaïveRats	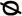	- No effect mechanical threshold - Paw pressure test	Walker et al., 2001a,b
	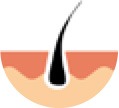		**L-AP4**		NaïveRats	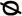	- No effect thermal withdrawal latency - Radiant heat source	Jin et al., 2009
			**L-AP4**	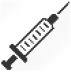	CAP injcRats	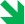	- No effect on paw withdrawal latency in acute pain - Attenuation of CAP-induced behavior - Flinching and lifting/licking/Radiant heat source	Govea et al., 2012
			**L-AP4**	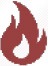	CarrageenanRats	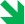	- Recovery of reduced weight load - Reduction of mechanical hyperalgesia - Von frey filaments/weight-bearing	Lee et al., 2013
		**Antagonist**	**MSOP**	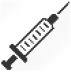	CAP injRats	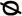	- No significant changes in withdrawal latencies - Radiant heat source	Jin et al., 2009
	**Spinal cord**	**Agonist**	**L-AP4**		NaïveRats	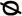	- No effect in spontaneous nociceptive behaviors - Elevating, shaking, stamping of the hindpaw/elevating or whipping of the tail/liking or biting the tail	Fisher and Coderre, 1996
	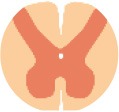		**L-AP4**	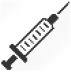	CAP injRats	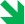	- Reduction of mechanical hypersensitivity, weak effect in thermal hyperalgesia - Von Frey/Paw immersion	Soliman et al., 2005
			**ACPT-I**	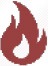	CarrageenanRats	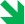	- Dose-dependent inhibition the nociceptive behavior - Paw pressure	Goudet et al., 2008
			**ACPT-I**	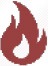	FormalinRats	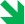	- No effect in naive animals - Dose-dependent inhibition the nociceptive behavior - Licking behavior	Goudet et al., 2008
			**ACPT-I**	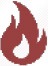	MARats	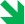	- Dose-dependent inhibition the nociceptive behavior - Paw pressure	Goudet et al., 2008
			**L-AP4**	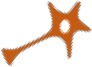	CCIRats	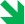	- Pretreatment produced reductions in the development of mechanical and cold hypersensitivity - Von Frey/1 cm deep 1°C water bath	Fisher et al., 2002
			**ACPT-I**	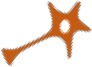	CCIRats	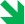	- Dose-dependent inhibition the nociceptive behavior - Paw pressure	Goudet et al., 2008
			**L-AP4**	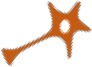	SNLRats	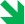	- Reduction of mechanical hypersensitivity - Von Frey	Chen and Pan, 2005
			**ACPT-I**	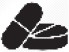	CIPNRats	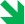	- Dose-dependent inhibition the nociceptive behavior - Paw pressure	Goudet et al., 2008
		**Antagonist**	**MAP4**		NaïveRats	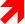	- Increase mechanical and thermal hypersensitivity - Radiant heat/Paw pressure	Chen and Pan, 2005
	**PAG**	**Agonist**	**L-SOP**		NaïveMice	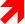	- Decrease the latency of the nociceptive reaction - Hot plate	Maione et al., 1998
	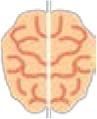		**L-SOP**	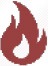	FormalinMice	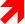	- Increase of phase II - Licking behavior	Maione et al., 2000
		**Antagonist**	**MSOP**		NaïveMice	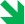	- Increase the latency of the nociceptive reaction - Hot plate	Maione et al., 1998

*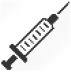 Symbols are used for model of pain induced by local injection, 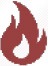 for inflammatory pain, 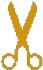 for post-operative pain, 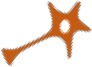
for neuropathic pain and 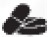 for chemotherapy-induced neuropathic pain models. 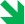, Decrease pain; 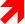, Increase pain; CAP, Capsaicin; CCI, Chronic constriction injury; CIPN, Chemotherapy-induced peripheral neuropathy; MA, Mono arthritis; SNL, Spinal nerve ligation*.

## Role of mGluR in Pain Transmission at the Spinal Cord Level

The spinal cord (SC) is the first relay in the transmission of sensory information from the periphery to the brain. It is submitted to control from peripheral inputs, interneurons within the SC and both inhibitory and excitatory descending pathways from supraspinal regions. This network makes the SC an important site for the modulation of signals generated at the periphery. Any alteration in neurons from the SC network can imbalance spinal relay and lead to chronic pain conditions.

The dorsal horn (DH) of the SC which receives nociceptive inputs is organized into different laminae, from the superficial laminae I to the deep laminae V. Most nociceptive fibers (Aδ- and C-fibers) superficially innervate laminae I-III and, to a lesser extent, laminae V, whereas low-threshold Aβ-fibers mainly project into laminae III–VI. Early studies have demonstrated that glutamate is released from primary afferent neurons into the DH in response to both acute and persistent painful stimuli, highlighting a role of the glutamatergic system in nociceptive transmission (Sluka and Westlund, 1992; Sorkin et al., 1992).

According to a recent single-cell RNA sequencing study of sensory neurons in the mouse DH, all mGluRs except mGlu6, are expressed within the spinal cord, the highest expression levels being measured for mGlu5 and 7 receptors (Häring et al., 2018). This high throughput data is in line with previous histological and pharmacological studies detailed below, and draw further attention to the relevance of targeting glutamate synapses for pain modulation in the dorsal horn of the spinal cord.

### Group I mGluRs

Immunoreactive cell bodies for group I mGluRs are widely spread throughout the superficial laminae of DH (Jia et al., 1999; Tang and Sim, 1999; Hudson et al., 2002). Intrathecal administration of group I mGluR agonists provokes hyperalgesia whereas group I mGluR antagonists induces analgesia in inflammatory and neuropathic pain models (Table 4) (Fisher and Coderre, 1996, 1998; Young et al., 1997; Fisher et al., 1998). Intrathecal injection of mGlu5 antagonist also reverses paclitaxel-induced neuropathic pain (Table 5; Xie et al., 2017). DH neuron excitability is increased after activation of spinal group I mGluRs in part due to due to inhibition of a voltage gated potassium channel (Hu et al., 2007). In line with this pharmacological evidence, knockdown or antibody approaches targeting mGlu1 receptor have demonstrated an antinociceptive effect in various pain models (Fundytus et al., 1998, 2001; Noda et al., 2003). Interestingly, recent studies have reported enhanced mGlu5 expression at the nuclear membrane in DH neurons after nerve injury. Using permeable mGlu5 antagonists reaching the cytoplasm, the authors have demonstrated that blocking intracellular mGlu5 had a greater antinociceptive effect than by blocking cell membrane expressed mGlu5 (Vincent et al., 2016). Pre-treatment with an excitatory amino acid transporter (EAAT) inhibitor, which is meant to decrease intracellular glutamate levels, decreases pain-related behavior in an inflammatory pain model (Vincent et al., 2017).

### Group II mGluRs

Among group II mGluRs, mGlu3 receptor is the most expressed in the DH, and its transcript is restricted to laminae II (Valerio et al., 1997; Berthele et al., 1999; Jia et al., 1999). However, only mGlu2 receptor expression appears to be enhanced in the SC (and DRG neurons) after administration of L-acetylcarnitine and histone deacetylase inhibitors, two compounds with antinociceptive properties, suggesting a greater role of spinal mGlu2 receptors in pain modulation (Chiechio et al., 2002, 2009). This discrepancy could be explained by expression pattern differences. Indeed, mGlu2 receptor is mostly pre-synaptic, while mGlu3 receptor is both pre- and post-synaptic (Nicoletti et al., 2011). Moreover, mGlu2 is expressed in microglia while mGlu3 is expressed in both microglia and astrocytes (Spampinato et al., 2018).

### Group III mGluRs

Transcripts of two group III members, mGlu4 and mGlu7 receptors, are detected in the spinal cord (Valerio et al., 1997). The expression of mGlu4 receptor is restricted to inner laminae II of the DH receiving nociceptive Aδ- and C-fibers inputs whereas mGlu7 receptor is expressed in both laminae I and II (Valerio et al., 1997; Vilar et al., 2013). In addition, the mGlu4 receptor may be expressed in spinal neurons, since its expression can still be observed after rhizotomy of the afferent fibers (Vilar et al., 2013). Activation of spinal group III mGluRs depletes glutamate release from primary afferents in nerve-injured rats (Table 7; Zhang et al., 2009). Furthermore, intrathecal administration of the group III broad-spectrum agonist L-AP4 reduces capsaicin-induced hypersensitivity and neuropathic pain symptoms (Fisher et al., 2002; Chen and Pan, 2005; Soliman et al., 2005). Intrathecal administration of the mGlu4 receptor PAM or agonist inhibits both inflammatory and neuropathic pain without altering acute pain thresholds in naive animals (Table 8; Goudet et al., 2008; Wang et al., 2011; Vilar et al., 2013). Conversely, the antiallodynic action of an mGlu4 agonist in inflammatory pain can be blocked by a photoswitchable mGlu4 NAM (Rovira et al., 2016). Positive allosteric modulation of spinal mGlu7 alleviates mechanical allodynia and thermal hyperalgesia induced by either carrageenan or skin incisions (Dolan et al., 2009). However, intrathecally administrated mGlu7 PAM has failed to relieve neuropathic pain (Wang et al., 2011). Both studies used the mGlu7 PAM named AMN082 (Mitsukawa et al., 2005). As mentioned earlier in the text, *in vivo*, AMN082 is rapidly metabolized and one of its metabolite inhibits several monoamine transporters (Sukoff Rizzo et al., 2011). Therefore, *in vivo* actions of AMN082 should be interpreted with caution since it may have multiple mode of action.

**Table 8:** Pain modulation following local administration of selective mGlu4, mGlu7 or mGlu8 ligands.

**Receptor subtype**	**Localization**	**Drugs type**	**Name**		**Models Species**	**Effects Tests**		**References**
**Group III**								
**∙ mGlu4**	**Spinal cord**	**Agonist**	**LSP4-2022**	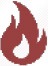	Carrageenan Mice	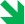	- Reduction of mechanical hypersensitivity - Von Frey	Vilar et al., 2013
	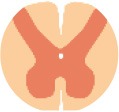		**LSP4-2022**	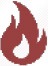	Carrageenan Rats	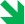	- Reduction of mechanical hypersensitivity - Paw pressure	Vilar et al., 2013
			**LSP4-2022**	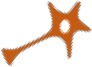	CCIRats	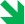	- No effect in naive animals - Reduction of mechanical hypersensitivity - Paw pressure	Vilar et al., 2013
		**PAM**	**PHCCC**	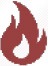	CarrageenanRats	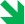	- No effect in naive animals - Dose-dependent inhibition the nociceptive behavior - Paw pressure	Goudet et al., 2008
			**PHCCC**	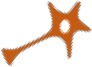	CCIRats	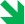	- No effect in naive animals - Dose-dependent inhibition the nociceptive behavior - Paw pressure	Goudet et al., 2008
			**VU0155041**	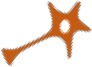	SNLRats	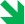	- Dose dependent attenuation of hyperalgesia - Von Frey/Paw immersion	Wang et al., 2011
	**Amygdala**	**Agonist**	**LSP4-2022**	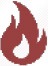	CFAMice	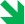	- Decrease mechanical allodynia and emotional components associated with chronic pain - Von Frey	Zussy et al., 2018
	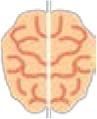	**PAM**	**Optogluram** Photoswitchable	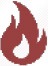	CFAMice	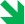	- Decrease mechanical allodynia and emotional components associated with chronic pain - Von Frey	Zussy et al., 2018
	**Striatum**	**PAM**	**VU0155041**	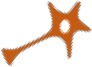	SNIRats	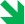	- No effect in both sham-operated and SNI rats - Tail flick	Rossi et al., 2013
**∙ mGlu7**	**Spinal cord** 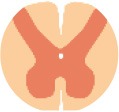	**PAM**	AMN082^*^	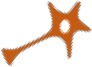	SNLRats	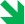	- No effect - Von Frey/Paw immersion	Wang et al., 2011
	**Amygdala**	**PAM**	AMN082^*^		NaïveRats	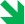	- Decrease mechanical threshold and increase of vocalizations - Knee compression	Palazzo et al., 2008
	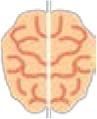		AMN082^*^	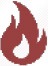	MARats	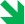	- No effect in mechanical threshold and vocalization - Knee compression	Palazzo et al., 2008
	**PAG**	**PAM**	AMN082^*^		NaïveMice	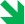	- Decrease mechanical threshold - Radiant heat source	Marabese et al., 2007
	**Striatum**	**PAM**	AMN082^*^		NaïveRats	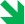	- Facilitation of pain - Dynamic Plantar Aesthesiometer/Tail Flick	Marabese et al., 2018
			AMN082^*^	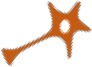	SNIRats	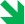	- Decrease mechanical allodynia and thermal hypersensitivity - Dynamic Plantar Aesthesiometer/Tail Flick	Marabese et al., 2018
		**NAM**	**ADX71743**	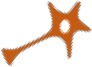	SNIRats	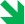	- No effect in mechanical threshold - Dynamic Plantar Aesthesiometer	Marabese et al., 2018
**∙ mGlu8**	**Amygdala**	**Agonist**	**DCPG**		NaïveRats	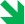	- No effect in mechanical threshold nor vocalization - Knee compression	Palazzo et al., 2008
			**DCPG**	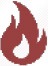	MARats	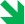	- Increase mechanical threshold and reduce vocalization - Knee compression	Palazzo et al., 2008
	**Striatum**	**Agonist**	**DCPG**	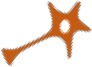	SNIRats		- Increase tail flick latency and mechanical threshold - No effect in sham animals - Tail flick/Von frey	Rossi et al., 2013
	**Striatum**	**PAM**	**AZ12216052**	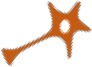	SNIRats	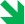	- Increase the tail flick latency - No effect in sham animals - Tail flick	Rossi et al., 2013
	**PAG**	**Agonist**	**DCPG**	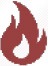	CarrageenanMice	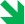	- Reduce pain behavior - Dynamic Plantar Aesthesiometer/Radiant heat source	Marabese et al., 2007
			**DCPG**	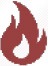	FormalinMice	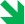	- Reduce pain behavior - Licking behavior	Marabese et al., 2007

* *Of note, in vivo actions of AMN082 should be interpreted with caution because they may involve other mechanisms in addition to mGlu7. Indeed, an AMN082 metabolite can inhibit monoamine transporters Sukoff Rizzo et al., 2011*.

*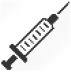 Symbols are used for model of pain induced by local injection, 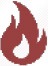 for inflammatory pain, 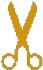 for post-operative pain, 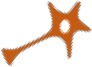 for neuropathic pain and 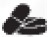 for chemotherapy-induced neuropathic pain models. 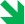, Decrease pain; 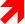, Increase pain; CCI, Chronic constriction injury; CFA, Complete Freund's Adjuvant; MA, Mono arthritis; SNL, Spinal nerve ligation; SNI, Spared nerve injury*.

## Contribution of mGluR to Supraspinal Mechanisms of Pain Perception

Integration of the nociceptive signal in the brain translates into a complex pain experience (Hunt and Mantyh, 2001). Pain processing in the supraspinal nervous system involves both ascending and descending pathways. Briefly, two main ascending pathways have been identified. The first one, the spinoparabrachial pathway, originates from the superficial dorsal horn and projects to areas of the brain concerned with affect: the parabrachial area (PB), the ventral medial nucleus (VMN) or the amygdala. The second one, the spinothalamic pathway, starts from the deep DH and projects to the thalamus and other areas of the cortex concerned with discrimination and affect. Different brain areas are involved in pain integration and processing. They are referred to as the pain matrix, a concept first described by Ronald Melzack in the late eighties (Melzack, 1990). It comprises several regions such as the primary and secondary sensorimotor cortex, insula, anterior cingulate cortex, thalamus, striatum, brainstem and cerebellum (Garcia-Larrea and Peyron, 2013). Descending pathways also involve high brain centers such as amygdala, hypothalamus and VMH, and nucleus in the midbrain and the brainstem, respectively, periaqueductal gray (PAG) and rostral ventromedial medulla (RVM).

mGluRs are widely express in neurons, astrocytes, oligodendrocytes, and microglia throughout the brain areas involved in pain processing. Consequently, there is an increasing interest in understanding the contribution of supraspinal mGluRs to pain modulation and many groups have investigated their potential for alleviating pain.

### Group I mGluRs

Although it is clearly established that activation of group I mGluRs at both the periphery and the spinal cord promotes pain, group I activation at the supraspinal level can elicit both antinociceptive and pronociceptive effects depending on the region investigated (Tables 4, 5). For instance, when applied in the amygdala, group I agonist promotes nociception (Li and Neugebauer, 2004; Kolber et al., 2010; Ren and Neugebauer, 2010; Tappe-Theodor et al., 2011). Reciprocally, stereotaxic injection of mGlu1 and mGlu5 receptor antagonists in the amygdala inhibits pain-related responses in a model of arthritic pain (Han and Neugebauer, 2005). Similarly, intra basolateral amygdala administration of group I mGluRs agonist alleviates inflammatory pain, an effect at least in part due to inhibition of prefrontal cortex neurons activity (Luongo et al., 2013). When applied to the thalamus, mGlu1 PAM potentiated nociceptive responses of thalamic neurons (Salt et al., 2014). Conversely, when administrated in the PAG, a region involved in modulation of the descending pain pathway, activation of group I mGluRs decreases the nociceptive response, likely through the inhibition of the GABAergic transmission (Maione et al., 2000; Drew and Vaughan, 2004). Moreover, PAG expressed mGlu5 contribute to the antinociceptive effect provoked by RVM cannabinoid receptor activation (de Novellis et al., 2005).

In an outstanding paper, authors used a selective photoactivable mGlu5 NAM enabling the precise spatiotemporal modulation of mGlu5 receptors to probe the involvement of thalamic mGlu5 receptors in pain processing. As expected, when injected systematically, the inactive caged compound has no effect on pain behavior of neuropathic animals. However, release of the active mGlu5 NAM by delivering light through implanted optical fibers in the ventrobasal thalamus, reduces neuropathic pain (Font et al., 2017).

An alternative photopharmacological strategy consists in using photoswitchable ligands that can be reversibly activated and inactivated by light (Goudet et al., 2018). This approach has been used to validate the role of amygdala-expressed mGlu5 in pain. A photoswitchable mGlu5 NAM has been injected locally in amygdala where it light-dependently reduced mechanical allodynia in a mice model of inflammatory pain (Gómez-Santacana et al., 2017), confirming previous preclinical studies (Han and Neugebauer, 2005).

Interestingly, global genetic disruption of mGlu5 in mice leads to increased basal mechanical withdrawal responses whereas conditional KO in the amygdala did not affect acute pain. However, both global and conditional KO prevent the establishment of mechanical hypersensitivity 180 min after formalin injection in the ipsi and contralateral paw (Kolber et al., 2010).

### Group II mGluRs

Accumulating evidence demonstrates that stimulation of group II mGluRs in supraspinal areas mediates analgesia (Table 6). Administration into the amygdala by microdialysis of group II agonist diminishes the response to noxious stimulation in an arthritis model of chronic pain (Li and Neugebauer, 2006). In the PAG, group II mGluR activation reinforces antinociceptive descending pathway (Maione et al., 2000). Local inhibition in the PAG or the RVM of the degradation of an endogenous peptide acting as an mGlu3 receptor agonist relieves pain in rat models of inflammatory and neuropathic pain (Yamada et al., 2012). However, studies have also reported a pronociceptive effect of CNS expressed group II mGluRs. For instance, blockage in the thalamus elicits antinociceptive effects, possibly via an inhibition of GABAergic inhibitory neurones (Neto and Castro-Lopes, 2000). Furthermore, microinjection of a group II agonist in the PAG induces pronociceptive effects by inhibiting descending pathway (Maione et al., 1998).

### Group III mGluRs

Broad range group III mGluR agonists were first used to elucidate the contribution of these receptors in pain processing in the CNS (Table 7). Early studies demonstrated that in the PAG a group III mGluR agonist facilitates pain related behavior (Maione et al., 1998, 2000), whereas in the amygdala group III agonist microinjection produces antinociceptive effects in an arthritis model (Li and Neugebauer, 2006). Development of more selective compounds for individual group III subtypes has allowed the more precise dissection of each members' contribution to nocifensive and affective pain responses within the CNS (Table 8). Of note, mGlu7 and mGlu8 have opposite effects in the PAG. Indeed, mGlu7 activation in PAG and amygdala is pronociceptive whereas mGlu8 activation is antinociceptive (Marabese et al., 2007; Palazzo et al., 2008). Similarly, in the nucleus tractus solitarius, mGlu7 activation has an antinociceptive effect on the cardiac-somatic reflex induced by pericardial capsaicin, while activation of mGlu8 receptors enhance cardiac nociception (Liu et al., 2012). Activation of mGlu7 in the nucleus accumbens by AMN082 has an antinociceptive effect and modulates relief learning (Kahl and Fendt, 2016). Blockade of mGlu7 in the PAG reduces the pain related behaviors in formalin and neuropathic pain models and differentially modulates RVM ON and OFF cell activity (Palazzo et al., 2013). Whereby, ON cells are neurons activated by noxious stimuli and inhibited by analgesics, and OFF cells are activated by analgesics and inhibited by painful stimuli (Palazzo et al., 2013).

Recently, dorsal striatum (DS) expressed mGlu7 receptors and their role in pain have been investigated. The DS is connected to the descending pain modulatory systems, including to the RVM. When locally administrated in the DS of sham animals, an mGlu7 PAM enhanced pain and simultaneously stimulates ON cells and inhibits OFF cells in the RVM. Whereas, in nerve-injured animals, the mGlu7 PAM has an anti-hyperalgesic effect in addition to increasing RVM OFF cell firing. This opposite effect of an mGluR7 PAM in acute or chronic pain conditions is assumed to be due to the recruitment of different pain pathways (Marabese et al., 2018). Interestingly, systemic administration of an mGluR7 PAM prevents the development of morphine tolerance (Gawel et al., 2018). A role of centrally expressed mGlu7 in epilepsy has also been reported (Sansig et al., 2001; Bertaso et al., 2008).

The first strong evidence of supraspinal mGlu4 involvement in pain processing is thanks to the recent development of an mGlu4 photoswitchable PAM allowing the time resolved control of endogenous receptors in freely behaving animals. Strikingly, dynamic modulation of mGlu4 receptor activation in the amygdala by the photoswitchable PAM reverses, in a light dependent manner, both inflammatory pain-related sensory and affective symptoms (Zussy et al., 2018). As compared to conventional compounds, this ligand enables precise temporal control of the mGlu4 receptor and, in contrast to optogenetics, allows endogenous receptor modulation, without the need of trangenesis. We expect that future development of photoswitchable ligands for other mGluRs will greatly improve our understanding of mGluRs in the pain neuraxis and co-morbidities associated with chronic pain conditions.

## Role of Glial mGluR in Pain

Beside neurons, mGluRs are also widely expressed in glial cells, noteworthy in microglia, astrocytes, and oligodendrocytes (for a recent review, see Spampinato et al., 2018). Astrocytes are the most abundant cell type in the brain, which are regulating neuronal function and remodeling synaptic structures. In addition to their physiological functions, astrocytes are involved in numerous diseases, such as chronic pain. Microglia act as resident macrophages, which function as sentinels of the CNS surveying potential damage. Following nerve injury, activated microglia surround the injured peripheral nerve terminals in the dorsal horn where they release different factors, such as brain-derived neurotrophic factor (BDNF), cytokines (TNFα, IL-1β, IL-6…) and glutamate, that will contribute to neuroinflammation, excitotoxicity and central sensitization. Numerous studies have shown that glial cells play a critical role in the development of neuropathic and inflammatory pain (Ji et al., 2013). For instance, microglia and astrocytes contribute to the central sensitization process that occurs in the setting of injury (Basbaum et al., 2009). Interestingly, all three groups of mGluRs are expressed in microglia and play a critical role in regulating microglial activity (Taylor et al., 2002, 2003; Byrnes et al., 2009; McMullan et al., 2012). *In vitro*, neuroinflammatory factors trigger an opposite regulation in the gene expression of the two predominant mGluR subtypes found in astrocytes and microglia, namely an upregulation of mGlu3 and a downregulation mGlu5 (Berger et al., 2012). Concerning group I mGluRs, activation of mGlu5 receptors inhibits microglial-associated inflammation and neurotoxicity (Byrnes et al., 2009), while little is known about mGlu1 receptors. Activation of group II mGluRs *in vitro* yields two opposite effects in cultured microglia, mGlu2 activation enhancing neurotoxicity whilst mGlu3 activation promotes neuroprotection (Taylor et al., 2002, 2005; Pinteaux-Jones et al., 2008). However, further studies are needed to understand the particular roles of these receptors, since activation of both mGlu2 and mGlu3 receptors have been reported to be neuroprotective *in vivo* (Fazio et al., 2018). Activation of group III mGluRs, notably mGlu4 receptors, reduces microglial reactivity (Taylor et al., 2003; Pinteaux-Jones et al., 2008; Ponnazhagan et al., 2016). Glial mGluRs modulate neuronal excitability and glutamate concentration in the synaptic and extrasynaptic regions (Pál, 2018). Of note, activation of group II and III, but not group I, attenuates export of glutamate from activated microglia through a cAMP-dependent mechanism (McMullan et al., 2012). Taken together, these results suggest that although less well studied than their neuronal counterparts, glial mGluRs may represent novel targets for the treatment of chronic pain.

## Conclusion

The growing number of selective compounds for the different mGluRs has significantly improved our understanding of the specific role of each subtype in nociception. Numerous evidences tend to suggest these receptors are promising targets for the treatment of chronic pain. However, at doses proven to be analgesic, mGlu1 antagonists are associated with motor and cognitive impairment (El-Kouhen et al., 2006; Zhu et al., 2008). Similarly, deficits in motor coordination phenotype has also been observed in mGlu1 conditional knockouts in the cerebellum (Nakao et al., 2007). Although mGlu5 antagonists may have psychoactive properties (Swedberg et al., 2014), mGlu5 blockade seems to elicit less side effects than mGlu1, suggesting that targeting mGlu5 may be more promising for the development of new analgesics. Regarding group II agonists, which have proven antinociceptive effects, a major concern for the treatment of persistent pain is the development of tolerance after repeated systematic injections (Jones et al., 2005; Zammataro et al., 2011). Nevertheless, epigenetic upregulation of endogenous mGlu2 receptor expression could counteract the drawback of tolerance. Group III metabotropic receptors are of a particular interest in drug development because their targeting may also decrease affective and cognitive disorders associated with chronic pain such as anxiety, depression, or fear (Zussy et al., 2018).

Given the analgesic effects observed after targeting peripheral mGluRs, peripherally restricted molecules may have satisfying analgesic effectiveness while decreasing the central-associated side effects. Furthermore, the use of new pharmacological tools such as photoswitchable or caged ligands, which allow the spatiotemporal tuning of mGluRs, could reduce off-target effects related to the modulation of the glutamatergic system outside the pain neuraxis.

## Author Contributions

All authors listed have made a substantial, direct and intellectual contribution to the work, and approved it for publication.

### Conflict of Interest Statement

The authors declare that the research was conducted in the absence of any commercial or financial relationships that could be construed as a potential conflict of interest.
